# Upregulation of AMPA receptor GluA1 phosphorylation by blocking adenosine A_1_ receptors in the male rat forebrain

**DOI:** 10.1002/brb3.1543

**Published:** 2020-01-29

**Authors:** Li‐Min Mao, John Q. Wang

**Affiliations:** ^1^ Department of Biomedical Sciences School of Medicine University of Missouri‐Kansas City Kansas City MO USA; ^2^ Department of Anesthesiology School of Medicine University of Missouri‐Kansas City Kansas City MO USA

**Keywords:** caudate putamen, DPCPX, GluA1, hippocampus, nucleus accumbens, phosphorylation, prefrontal cortex, RRID: AB_2113602, RRID: AB_2492127, RRID: AB_2492128, RRID: AB_476693, RRID: nif‐0000 ‐30467

## Abstract

**Objective:**

The adenosine A_1_ receptor is a G_αi/o_ protein‐coupled receptor and inhibits upon activation cAMP formation and protein kinase A (PKA) activity. As a widely expressed receptor in the mammalian brain, A_1_ receptors are implicated in the modulation of a variety of neuronal and synaptic activities. In this study, we investigated the role of A_1_ receptors in the regulation of α‐amino‐3‐hydroxy‐5‐methyl‐4‐isoxazolepropionic acid (AMPA) receptors in the adult rat brain in vivo.

**Methods:**

Adult male Wistar rats were used in this study. After a systemic injection of the A_1_ antagonist DPCPX, rats were sacrificed and several forebrain regions were collected for assessing changes in phosphorylation of AMPA receptors using Western blots.

**Results:**

A systemic injection of the A_1_ antagonist DPCPX induced an increase in phosphorylation of AMPA receptor GluA1 subunits at a PKA‐dependent site, serine 845 (S845), in the two subdivisions of the striatum, the caudate putamen, and nucleus accumbens. DPCPX also increased S845 phosphorylation in the medial prefrontal cortex (mPFC) and hippocampus. The DPCPX‐stimulated S845 phosphorylation was a transient and reversible event. Blockade of G_αs/olf_‐coupled dopamine D_1_ receptors with a D_1_ antagonist SCH23390 abolished the responses of S845 phosphorylation to DPCPX in the striatum, mPFC, and hippocampus. DPCPX had no significant impact on phosphorylation of GluA1 at serine 831 and on expression of total GluA1 proteins in all forebrain regions surveyed.

**Conclusion:**

These data demonstrate that adenosine A_1_ receptors maintain an inhibitory tone on GluA1 S845 phosphorylation under normal conditions. Blocking this inhibitory tone leads to the upregulation of GluA1 S845 phosphorylation in the striatum, mPFC, and hippocampus via a D_1_‐dependent manner.

## Graphical abstract

Our data demonstrate that adenosine A_1_ receptors maintain an inhibitory tone on GluA1 S845 phosphorylation under normal conditions. Blocking this inhibitory tone leads to the upregulation of GluA1 S845 phosphorylation in the striatum, mPFC, and hippocampus via a D_1_‐dependent manner.

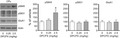

## SIGNIFICANCE

In this study, we found that an A_1_ receptor antagonist DPCPX increased phosphorylation of AMPA receptor GluA1 subunits in the key limbic reward regions, including the striatum, medial prefrontal cortex, and hippocampus. Blocking dopamine D_1_ receptors abolished the responses of GluA1 phosphorylation to DPCPX. These data reveal an inhibitory linkage from adenosine A_1_ receptors to AMPA receptors. Malfunction of this linkage may be linked to various psychiatric and neurological disorders such as drug addiction and anhedonic depression.

## 1 INTRODUCTION

The α‐amino‐3‐hydroxy‐5‐methyl‐4‐isoxazolepropionic acid (AMPA) receptor is widely expressed in the mammalian brain and is actively involved in the regulation of a variety of cellular and synaptic activities (Bernard, Somogyi, & Bolam, 1997; Kondo, Okabe, Sumino, & Okado, 2000; Reimers, Milovanovic, & Wolf, 2011). Like other glutamate receptors, AMPA receptors are tightly regulated by a phosphorylation‐dependent mechanism (Lu & Roche, 2012). Through protein kinase C and Ca^2+^/calmodulin‐dependent protein kinase II, one of four AMPA receptor subunits, GluA1 (also known as GluR1; Greger, Ziff, & Penn, 2007), is phosphorylated at an intracellular C‐terminal site, serine 831 (S831). In addition, GluA1 is phosphorylated at serine 845 (S845) by protein kinase A (PKA; Barria, Derkach, & Soderling, 1997; Mammen, Kameyama, Roche, & Huganir, 1997; Roche, O'Brien, Mammen, Bernhardt, & Huganir, 1996; Serulle et al., 2007). It is clear now that phosphorylation of GluA1 at these sites is critical for regulating GluA1/AMPA receptors in a set of their properties in neurons and transfected cells (Wang et al., 2014).

Many neurotransmitters regulate GluA1 in its phosphorylation status. Among them is dopamine which has been extensively studied in dopamine responsive brain regions, especially the mesolimbic reward circuit. Within the striatum, dopamine D_1_ and D_2_ receptors are highly expressed and are segregated into two subpopulations of medium spiny projection neurons, that is, D_1_ and D_2_ receptors in respective striatonigral and striatopallidal neurons (Aubert, Ghorayeb, Normand, & Bloch, 2000; Bertran‐Gonzalez, Herve, Girault, & Valjent, 2010; Gerfen et al., 1990). Consistent evidence shows that pharmacological activation of G_αs/olf_‐coupled D_1_ receptors by the direct or indirect D_1_ agonists upregulated GluA1 phosphorylation at the PKA site (S845) in the striatum, probably via a signaling mechanism involving the cAMP/PKA pathway (Chao, Lu, Lee, Huganir, & Wolf, 2002; Mao, Diaz, Fibuch, & Wang, 2013; Price, Kim, & Raymond, 1999; Snyder et al., 2000; Swayze, Lise, Levinson, Phillips, & El‐Husseini, 2004; Xue et al., 2014, 2017). In contrast, G_αi/o_‐coupled D_2_ receptors are negatively coupled to GluA1 in its S845 phosphorylation as the D_2_ antagonist enhanced S845 phosphorylation (Hakansson et al., 2006; Xue et al., 2017).

The purine nucleotide adenosine is a ubiquitous neuromodulator which interacts with four subtypes of adenosine receptors (A_1_, A_2A_, A_2B_, and A_3_; Fredholm, 2011; Fredholm, Ijzerman, Jacobson, Klotz, & Linden, 2001; Sheth, Brito, Mukherjea, Rybak, & Ramkumar, 2014). Within the brain, A_1_ and A_2A_ subtypes are primarily expressed (Sheth et al., 2014). The former is coupled to G_αi/o_ proteins, whereas the latter is coupled to G_αs/olf_ proteins. Thus, activation of A_1_ and A_2A_ receptors inhibits and stimulates adenylyl cyclase and the cAMP/PKA pathway, respectively (Fredholm et al., 2001; Kull, Svenningsson, & Fredholm, 2000; Sheth et al., 2014). Within the striatum, A_1_ receptors are noticeably colocalized with D_1_ receptors in striatonigral neurons (Fuxe, Ferre, Genedani, Franco, & Agnati, 2007; Fuxe et al., 2010). As such, a functional A_1_‐D_1_ receptor–receptor interaction was suggested in these postsynaptic neurons (Ferre et al., 1994; Fuxe et al., 2007, 2010). With regard to the linkage to AMPA receptors, dopamine D_1_ signaling has been well characterized to be positively coupled to GluA1 S845 phosphorylation as aforementioned. However, whether A_1_ receptors regulate S845 phosphorylation has been less studied to date (Chen et al., 2014; Hobson et al., 2013; Stockwell, Chen, Niazi, Nosib, & Cayabyab, 2016).

In this study, we attempted to evaluate the role of A_1_ receptors in the regulation of GluA1 phosphorylation and expression in the adult rat brain in vivo. To achieve this, we investigated the effect of an A_1_ antagonist 8‐cyclopentyl‐1,3‐dipropylxanthine (DPCPX) on GluA1 phosphorylation at S845 and S831 sites in different brain regions, including the caudate putamen (CPu), nucleus accumbens (NAc), medial prefrontal cortex (mPFC), and hippocampus. We then tested the effect of DPCPX on GluA1 phosphorylation in the presence of a dopamine D_1_ antagonist SCH23390.

## 2 MATERIALS AND METHODS

### 2.1 Animals

We used male rats in this study. Adult Wistar rats (2–3 months, 310–380 g) were purchased from a vendor (Charles River) and were kept at 12‐hr light/12‐hr dark cycle and 23°C with water and food available ad libitum. At 5–6 days after habituation, animals were used for experiments. The protocol of animal use and care was approved by the Institutional Animal Care and Use Committee.

### 2.2 Drug administration and experimental arrangements

All drugs were administered via intraperitoneal (i.p.) injections. We calculated doses of drugs as their salt form. All drugs were injected in a volume of 1 ml/kg. Age‐matched rats were injected with a vehicle solution and served as controls. A series of experiments were conducted with rats randomly divided into different groups using a computer‐generated randomization table (GraphPad software/QuickCalcs). Size of sample was determined by the sample size calculation (*α* = .05, *β* = .2; 80% power). There were no differences in sample size between the beginning and end of the experiments. We based the criteria for inclusion/exclusion on the health state of animals. The healthy animals that showed no sign of illness as evaluated by the body weight and visual observations were used in the analysis.

We first investigated the effect of DPCPX, an A_1_ antagonist which was approximately 1,000‐fold more potent at A_1_ than A_2A_ receptors in the striatum (Fredholm & Lindstrom, 1999). Three groups of rats (*n* = 4 per group) were subjected to an i.p. injection of vehicle or DPCPX at either 0.25 or 2.5 mg/kg and were then sacrificed 20 min after DPCPX injection. Changes in GluA1 phosphorylation and expression in four different brain regions, including the CPu, NAc, mPFC, and hippocampus, were subsequently assayed using Western blots. Doses of DPCPX (0.25 and 2.5 mg/kg) were selected because an i.p. injection of DPCPX at 3 mg/kg blocked the anxiolytic‐like response to the A_1_ selective agonist and positive allosteric modulator (Prediger, Silva, Batista, Bittencourt, & Takahashi, 2006; Vincenzi et al., 2016). Second, we conducted a time course study. Following an injection of vehicle or DPCPX (2.5 mg/kg, i.p.), rats were sacrificed at different time points (1, 3, and 6 hr) for analyzing changes in GluA1 phosphorylation and expression in different brain regions. Two groups of rats (*n* = 4 per group) were used at each time point. Finally, the effect of DPCPX was tested in the presence of a dopamine D_1_ receptor antagonist in four groups of rats (*n* = 4 per group). The D_1_ antagonist SCH23390 (0.1 mg/kg, i.p.) was administered 10 min prior to DPCPX (2.5 mg/kg, i.p.). Rats were sacrificed 15–20 min after DPCPX injection for immunoblot analysis of changes in GluA1 phosphorylation in different brain regions.

### 2.3 Western blot

To extract proteins from brain tissue, rats were anesthetized, followed by decapitation and removal of brains. Removed brains were frozen in isopentane cooled on dry ice. Brains were cut into coronal sections using a razor blade. The brain regions of interest, including the CPu, NAc, mPFC, and hippocampus, were dissected from the sections. The mPFC contained the anterior cingulate, prelimbic and infralimbic cortex (Heidbreder & Groenewegen, 2003). In a radioimmunoprecipitation assay buffer containing 20 mM Tris‐HCl, pH 7.5, 150 mM NaCl, 1 mM Na_2_EDTA, 1 mM EGTA, 1% NP‐40, 1% sodium deoxycholate, 2.5 mM sodium pyrophosphate, 1 mM β‐glycerophosphate, 1 mM Na_3_VO_4_, and 1 µg/ml leupeptin (Cell Signaling Technology), the dissected brain tissue was homogenized. Homogenates were centrifuged at 800 *g* (10 min). Protein concentration of the supernatant was determined. Samples were then used for Western blot which was performed as described previously (Mao et al., 2013; Mao, Faris, & Wang, 2018). Briefly, on NuPAGE Novex 4%–12% gels (Invitrogen), proteins (32 µg/well) in brain lysates were separated. Separated proteins on gels were transferred to membranes (polyvinylidene fluoride). The membranes were blocked, followed by incubation with a primary antibody overnight at 4°C. A secondary antibody linked to horseradish peroxidase was incubated to interact with the primary antibody. Immunoblots on membranes were visualized using an enhanced chemiluminescence reagent. To quantitatively analyze immunoblots, we measured optical density of blots using analysis software (NIH ImageJ, RRID: nif‐0000‐30467). All values of optical density were normalized to β‐actin.

### 2.4 Antibodies

All primary antibodies used in this study and their characterizations are listed in Table 1. The rabbit antibodies against the phosphorylated GluA1 subunit include the antibody against phospho‐S831 (pS831, PhosphoSolutions; cat. #: p1160‐831; RRID: AB_2492127) or phospho‐S845 (pS845, PhosphoSolutions; cat. #: 1160‐845; RRID: AB_2492128). In addition, the rabbit antibody against GluA1 (Millipore; cat. #: AB1504; RRID: AB_2113602) or β‐actin (Sigma‐Aldrich; cat. #: A2066; RRID: AB_476693) was used.

**Table 1:** Primary antibodies used

Antigen	Description of immunogen	Source, host species, catalog No., clone or lot No., RRID	Concentration used (µg/ml)	Primary antibody blocking solution
β‐Actin	C‐terminal actin fragment: Ser‐Gly‐Pro‐Ser‐Ile‐Val‐His‐Arg‐Lys‐Cys‐Phe	Sigma‐Aldrich, rabbit polyclonal, A2066, RRID: AB_476693	0.2	3% Nonfat milk‐PBS 0.1% Tween‐20
GluA1	A KLH‐conjugated peptide corresponding to human GluA1 at the cytoplasmic domain	EMD Millipore, rabbit polyclonal, AB1504, RRID: AB_2113602	1	3% Nonfat milk‐PBS 0.1% Tween‐20
pS831	A phosphopeptide corresponding to residues surrounding the phospho‐Ser831 of GluA1	PhosphoSolutions, rabbit polyclonal, p1160‐831, RRID: AB_2492127	1	3% Nonfat milk‐PBS 0.1% Tween‐20
pS845	A phosphopeptide corresponding to residues surrounding the phospho‐Ser845 of GluA1	PhosphoSolutions, rabbit polyclonal, p1160‐845, RRID: AB_2492128	1	3% Nonfat milk‐PBS 0.1% Tween‐20

### 2.5 Pharmacological agents

Pharmacological agents include DPCPX (Tocris) and R(+)‐SCH23390 hydrochloride (Sigma‐Aldrich). We dissolved DPCPX in dimethyl sulfoxide (DMSO) and 0.1 M sodium hydroxide. Stock solutions of DPCPX were diluted in saline. Final concentrations of DMSO and sodium hydroxide were 15% (v/v) and 8% (v/v), respectively (Uzbay, Kayir, & Ceyhan, 2007). SCH23390 was dissolved in physiological saline. All drugs were prepared freshly at the day of experiments.

### 2.6 Statistics

Data were statistically analyzed following tests for the normality of data. They are expressed as means ± *SEM*. In this study, no test for outliers was conducted on the data. No rats were excluded from the analysis. As appropriate, a two‐tailed unpaired Student's *t* test or one‐ or two‐way analysis of variance (ANOVA) with a multiple comparison post hoc test was conducted to analyze data. The statistical significance was determined by a *p* value < .05.

## 3 RESULTS

### 3.1 Effects of the A_1_ antagonist DPCPX on GluA1 phosphorylation

**Figure 1:**
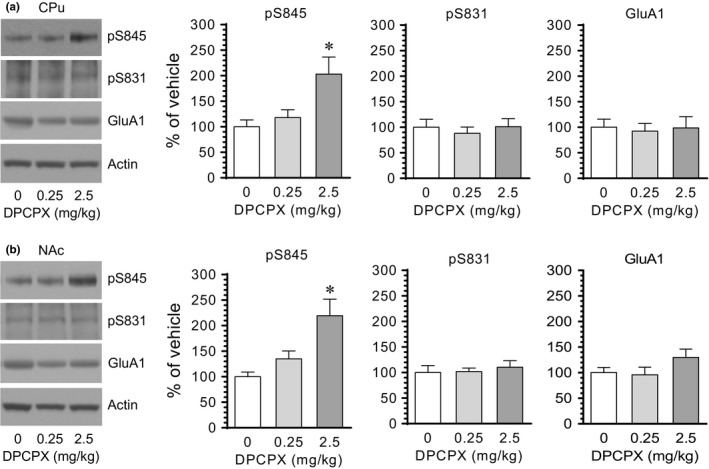
The effect of DPCPX on GluA1 phosphorylation in the rat CPu and NAc. (a) The effect of DPCPX on GluA1 phosphorylation and expression in the CPu. (b) The effect of DPCPX on GluA1 phosphorylation and expression in the NAc. Note that DPCPX at a higher dose (2.5 mg/kg) but not a lower dose (0.25 mg/kg) induced an increase in pS845 levels in both the CPu and NAc. Representative immunoblots are shown left to the quantified data. Rats were administered with vehicle or DPCPX at either 0.25 or 2.5 mg/kg (i.p.) and were then sacrificed 20 min after drug administration for subsequent analysis of changes in S845 and S831 phosphorylation using Western blots. Data are presented as means ± *SEM* (*n* = 4 per group) and were analyzed using one‐way ANOVA: CPu‐pS845, *F*(2,9) = 6.15, *p* = .021; CPu‐pS831, *F*(2,9) = 0.25, *p* = .785; CPu‐GluA1, *F*(2,9) = 0.05, *p* = .949; NAc‐pS845, *F*(2,9) = 8.29, *p* = .009; NAc‐pS831, *F*(2,9) = 0.23, *p* = .801; and NAc‐GluA1, *F*(2,9) = 1.79, *p* = .222. *p < .05 versus vehicle

To determine the impact of blockade of A_1_ receptors on GluA1 phosphorylation, we subjected rats to a single i.p. injection of the A_1_ antagonist DPCPX. We sacrificed rats 20 min after DPCPX injection to assay changes in GluA1 phosphorylation at S845 and S831 in different brain regions using Western blots. In the CPu, DPCPX at a lower dose (0.25 mg/kg) had no significant effect on GluA1 phosphorylation at S845 and S831 (Figure 1a). Noticeably, at a higher dose (2.5 mg/kg), DPCPX induced a marked increase in pS845 levels, while DPCPX did not alter pS831 levels. Cellular levels of total GluA1 proteins remained stable in response to DPCPX. Similar results were observed in the NAc. As shown in Figure 1b, DPCPX at 2.5 although not at 0.25 mg/kg elevated pS845 levels in this region. DPCPX at either dose had a minimal impact on S831 phosphorylation and total GluA1 expression. These results indicate that pharmacological blockade of adenosine A_1_ receptors with DPCPX upregulates GluA1 phosphorylation selectively at S845 in the two subdivisions of the striatum (CPu and NAc).

**Figure 2:**
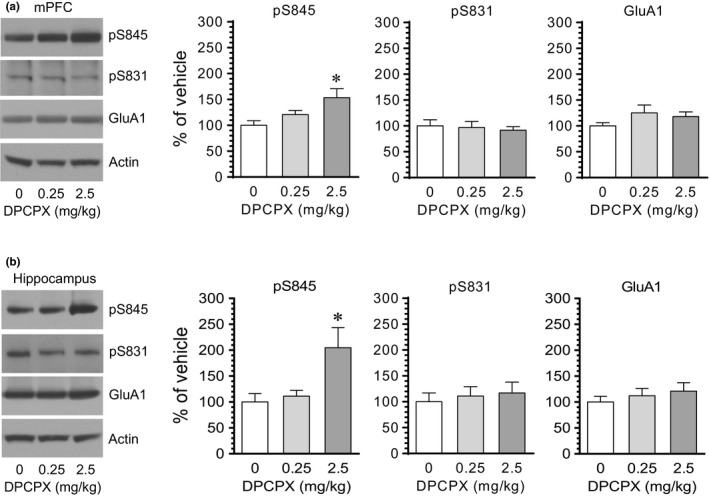
The effect of DPCPX on GluA1 phosphorylation in the rat mPFC and hippocampus. (a) The effect of DPCPX on GluA1 phosphorylation and expression in the mPFC. (b) The effect of DPCPX on GluA1 phosphorylation and expression in the hippocampus (Hippo). Note that DPCPX at a higher dose (2.5 mg/kg) but not a lower dose (0.25 mg/kg) induced an increase in pS845 levels in the mPFC and hippocampus. Representative immunoblots are shown left to the quantified data. Rats were administered with vehicle or DPCPX at either 0.25 or 2.5 mg/kg (i.p.) and were then sacrificed 20 min after drug administration for subsequent analysis of changes in S845 and S831 phosphorylation using Western blots. Data are presented as means ± *SEM* (*n* = 4 per group) and were analyzed using one‐way ANOVA: mPFC‐pS845, *F*(2,9) = 4.92, *p* = .036; mPFC‐pS831, *F*(2,9) = 0.17, *p* = .844; mPFC‐GluA1, *F*(2,9) = 1.38, *p* = .300; hippocampus‐pS845, *F*(2,9) = 5.27, *p* = .031; hippocampus‐pS831, *F*(2,9) = 0.22, *p* = .804; and hippocampus‐GluA1, *F*(2,9) = 0.54, *p* = .599. *p < .05 versus vehicle

We next examined the effect of DPCPX on GluA1 phosphorylation in the mPFC and hippocampus. DPCPX at 2.5 but not at 0.25 mg/kg (20 min prior to tissue collection) elevated the pS845 level in the mPFC (Figure 2a). Like the mPFC, the hippocampus showed a change in GluA1 S845 phosphorylation in response to DPCPX. After an injection of DPCPX at 2.5 but not at 0.25 mg/kg, the pS845 level in the hippocampus was elevated in DPCPX‐treated rats compared to vehicle‐treated rats (Figure 2b). Little change in pS831 and total GluA1 levels was seen in the two regions following DPCPX administration at either dose. These data indicate that DPCPX was able to enhance S845 phosphorylation in the mPFC and hippocampus.

### 3.2 A time course study of the DPCPX effect

**Figure 3:**
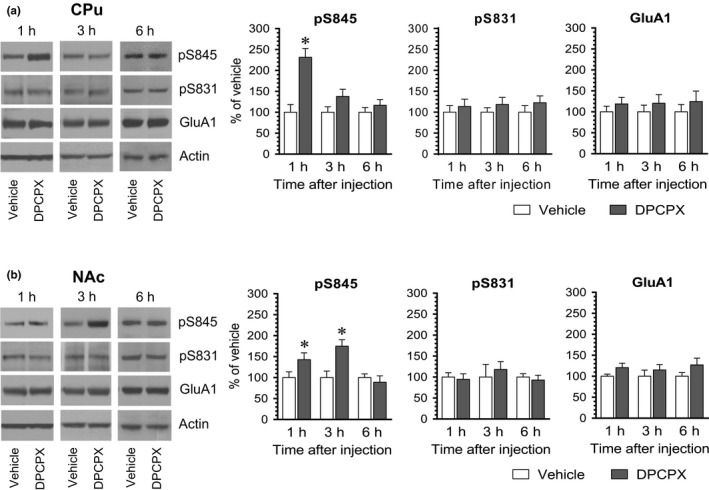
The time‐dependent effect of DPCPX on GluA1 phosphorylation in the rat CPu and NAc. (a) The time‐dependent effect of DPCPX on GluA1 phosphorylation in the CPu. (b) The time‐dependent effect of DPCPX on GluA1 phosphorylation in the NAc. Note that DPCPX induced a reversible increase in pS845 levels in both the CPu and NAc. Representative immunoblots are shown left to the quantified data. Rats received an i.p. injection of vehicle or DPCPX (2.5 mg/kg) and were then sacrificed at a different time point (1, 3, or 6 hr) after drug administration for subsequent analysis of changes in S845 and S831 phosphorylation using Western blots. Data are presented as means ± *SEM* (*n* = 4 per group) and were analyzed using Student's *t* test: CPu‐pS845: *p* = .004 (1 hr), *p* = .130 (3 hr), and *p* = .381 (6 hr); and NAc‐pS845: *p* = .039 (1 hr), *p* = .014 (3 hr), and *p* = .554 (6 hr). *p < .05 versus vehicle at the same time point

We next carried out a time course study to define temporal properties of the DPCPX effect. To this end, we injected vehicle or DPCPX at an effective dose (2.5 mg/kg) to rats. We then sacrificed rats at different time points (1, 3, and 6 hr) after drug injection and analyzed changes in S845 and S831 phosphorylation in different brain regions. Like the positive effect of DPCPX observed at 20 min from the above studies, DPCPX at 1 hr elevated pS845 levels in the CPu (Figure 3a). This elevation became insignificant at 3 hr. At 6 hr, no significant difference in pS845 levels was found between DPCPX‐ and vehicle‐treated groups. In the NAc, a significant increase in pS845 levels was seen at 1 hr in DPCPX‐treated rats compared to vehicle‐treated rats (Figure 3b). This increase persisted at 3 hr and declined to a level insignificantly different from the value obtained from the vehicle group at 6 hr. At all time points surveyed, DPCPX induced minimal changes in pS831 and GluA1 levels in the CPu and NAc. Thus, DPCPX generally induced a short‐lived and reversible increase in GluA1 S845 phosphorylation in the striatum.

**Figure 4:**
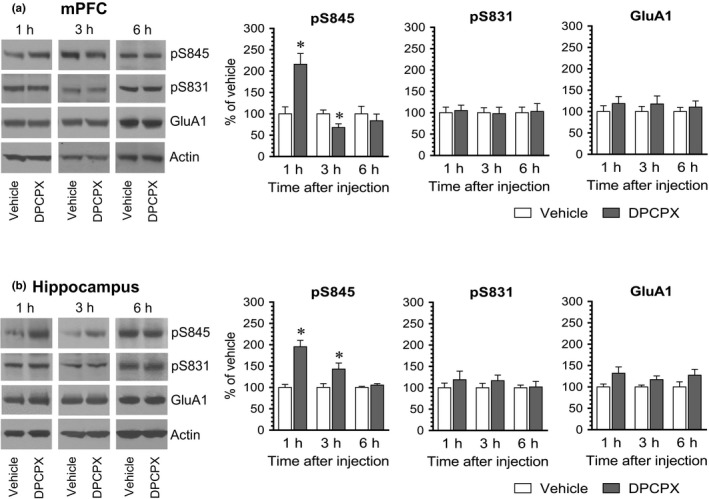
The time‐dependent effect of DPCPX on GluA1 phosphorylation in the rat mPFC and hippocampus. (a) The time‐dependent effect of DPCPX on GluA1 phosphorylation in the mPFC. (b) The time‐dependent effect of DPCPX on GluA1 phosphorylation in the hippocampus. Note that DPCPX induced a biphasic effect in the mPFC, that is, an increase followed by a decrease in pS845 levels. Representative immunoblots are shown left to the quantified data. Rats received an i.p. injection of vehicle or DPCPX (2.5 mg/kg) and were then sacrificed at a different time point (1, 3, or 6 hr) after drug administration for subsequent analysis of changes in S845 and S831 phosphorylation using Western blots. Data are presented as means ± *SEM* (*n* = 4 per group) and were analyzed using Student's *t* test: mPFC‐pS845: *p* = .006 (1 hr), *p* = .022 (3 hr), and *p* = .522 (6 hr); and hippocampus‐pS845: *p* = .001 (1 hr), *p* = .040 (3 hr), and *p* = .220 (6 hr). *p < .05 versus vehicle at the same time point

We also examined the effect of DPCPX at different time points in the mPFC and hippocampus. In the mPFC, pS845 levels were substantially enhanced at 1 hr following DPCPX administration (Figure 4a). Noticeably, pS845 levels in this region were reduced at 3 hr in DPCPX‐treated rats relative to vehicle‐treated rats. At 6 hr, an insignificant change in pS845 levels was induced by DPCPX. Thus, it appears that DPCPX induces a biphasic pattern of changes in S845 phosphorylation in the mPFC, that is, an initial increase followed by a decrease in pS845 levels. In the hippocampus, DPCPX stimulated S845 phosphorylation at 1 and 3 hr, while DPCPX did not at 6 hr (Figure 4b), indicating that DPCPX induces a dynamic and reversible increase in S845 phosphorylation in the hippocampus. Additionally, DPCPX had no significant effect on S831 phosphorylation and GluA1 expression in the mPFC and hippocampus at three time points surveyed.

### 3.3 Effects of the D_1_ antagonist on the DPCPX‐induced GluA1 phosphorylation

**Figure 5:**
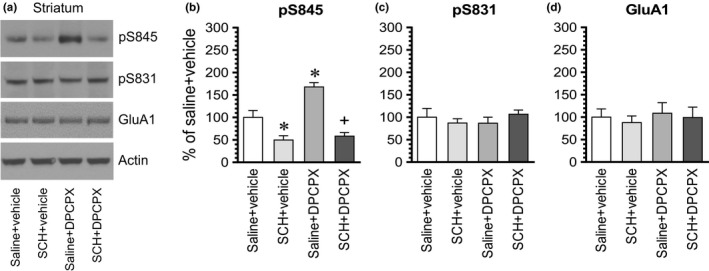
The effect of SCH23390 on the DPCPX‐induced GluA1 S845 phosphorylation in the rat striatum. (a) Representative immunoblots illustrating the effect of SCH23390 on the DPCPX‐induced S845 phosphorylation in the striatum. (b–d) Quantifications of the effect of SCH23390 on the DPCPX‐induced S845 phosphorylation in the striatum. Note that SCH23390 reduced basal S845 phosphorylation and completely blocked the DPCPX‐induced S845 phosphorylation. Rats were administered with SCH23390 (SCH, 0.1 mg/kg, i.p.) 10 min prior to DPCPX (2.5 mg/kg, i.p.) and were then sacrificed 15–20 min after DPCPX administration for subsequent analysis of changes in S845 and S831 phosphorylation using Western blots. Data are presented as means ± *SEM* (*n* = 4 per group) and were analyzed using two‐way ANOVA: pS845: saline versus SCH23390, *F*(1,12) = 51.72, *p* < .001, vehicle versus DPCPX, *F*(1,12) = 11.99, *p* = .005, and interaction, *F*(1,12) = 7.17, *p* = .020. *p < .05 versus saline + vehicle. ^+^ *p* < .05 versus saline + DPCPX

To determine whether blockade of the dopamine D_1_ receptor has any effect on the DPCPX‐induced GluA1 phosphorylation, we administered a D1 antagonist SCH23390 (0.1 mg/kg, i.p.) 10 min prior to an i.p. injection of DPCPX at an effective dose of 2.5 mg/kg. Rats were sacrificed 15–20 min after DPCPX administration for immunoblot analysis of changes in GluA1 phosphorylation and expression. In the striatum, SCH23390 alone reduced the basal level of pS845 signals, while DPCPX alone elevated it as expected (Figure 5a,b). Remarkably, SCH23390 completely blocked the increase in pS845 levels induced by DPCPX. No significant change in pS831 and GluA1 levels was seen in all drug‐treated groups (Figure 5c,d). These results indicate that the A_1_ antagonist DPCPX increases GluA1 S845 phosphorylation in striatal neurons via a D_1_‐dependent mechanism.

**Figure 6:**
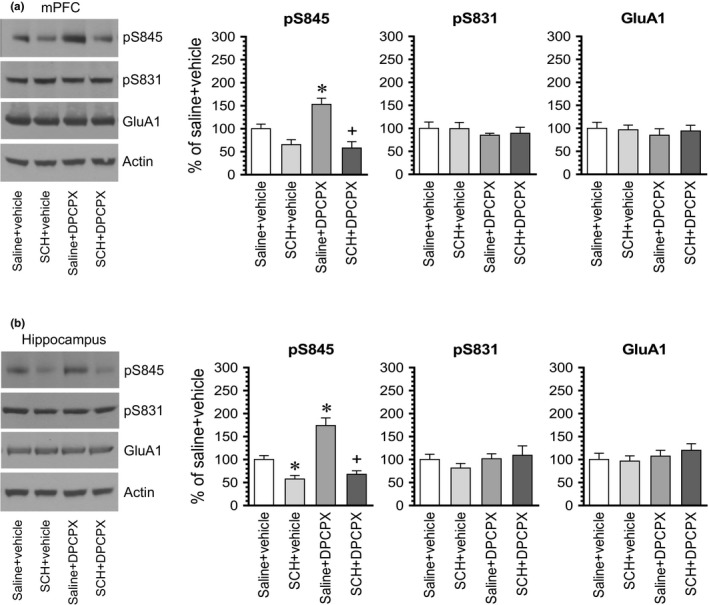
The effect of SCH23390 on DPCPX‐induced GluA1 S845 phosphorylation in the rat mPFC and hippocampus. (a) The effect of SCH23390 on DPCPX‐induced S845 phosphorylation in the mPFC. (b) The effect of SCH23390 on DPCPX‐induced S845 phosphorylation in the hippocampus (Hippo). Note that SCH23390 completely blocked the DPCPX‐induced S845 phosphorylation in the two regions. Representative immunoblots are shown left to the quantified data. Rats were administered with SCH23390 (SCH, 0.1 mg/kg, i.p.) 10 min prior to DPCPX (2.5 mg/kg, i.p.) and were then sacrificed 15–20 min after DPCPX administration for subsequent analysis of changes in S845 and S831 phosphorylation using Western blots. Data are presented as means ± *SEM* (*n* = 4 per group) and were analyzed using two‐way ANOVA: mPFC‐pS845: saline versus SCH23390, *F*(1,12) = 33.01, *p* < .001, vehicle versus DPCPX, *F*(1,12) = 4.14, *p* = .064, and interaction, *F*(1,12) = 7.18, *p* = .020; and hippocampus‐pS845: saline versus SCH23390, *F*(1,12) = 59.25, *p* < .001, vehicle versus DPCPX, *F*(1,12) = 18.87, *p* = .001, and interaction, *F*(1,12) = 10.74, *p* = .007. *p < .05 versus saline + vehicle. ^+^ *p* < .05 versus saline + DPCPX

In the mPFC, DPCPX elevated S845 signals (Figure 6a). However, DPCPX failed to increase S845 phosphorylation in the presence of SCH23390. The pS845 level in the group of rats treated with SCH23390 + DPCPX was not statistically different from that seen in the group of rats treated with SCH23390 + vehicle. Similarly, S845 phosphorylation was not altered by DPCPX in the presence of SCH23390 in the hippocampus. As shown in Figure 6b, while DPCPX alone increased pS845 levels, DPCPX did not induce an increase in pS845 levels in rats pretreated with SCH23390 compared to rats treated with SCH23390 + vehicle. GluA1 phosphorylation at S831 was resistant to SCH23390, DPCPX, or combination of two drugs in the mPFC and hippocampus. GluA1 expression remained stable in response to all drug treatments in the two regions. Thus, like the striatum, the basal activity of dopamine D_1_ receptors is required for the A_1_ antagonist‐induced GluA1 S845 phosphorylation in both the mPFC and hippocampus.

## 4 DISCUSSION

We initiated an effort to investigate the role of adenosine A_1_ receptors in the regulation of GluA1 phosphorylation and expression in the adult rat forebrain in vivo. We found that a systemic injection of the A_1_ antagonist DPCPX induced a significant increase in GluA1 phosphorylation at S845 in multiple brain regions, including the CPu, NAc, mPFC, and hippocampus. The increase in GluA1 phosphorylation induced by DPCPX was time‐dependent and reversible. Pretreatment with a dopamine D_1_ antagonist SCH23390 blocked the response of S845 phosphorylation to DPCPX in the striatum, mPFC, and hippocampus. In contrast to S845, GluA1 phosphorylation at S831 was not altered by DPCPX in all four brain regions. These data indicate that A_1_ receptors exert tonic inhibition of GluA1 S845 phosphorylation in striatal, cortical, and hippocampal neurons under normal conditions. Blocking this tonic inhibition induces the D_1_‐dependent upregulation of S845 phosphorylation.

Dopamine D_1_ receptors are selectively expressed in striatonigral output neurons in the striatum (Aubert et al., 2000; Bertran‐Gonzalez et al., 2010; Gerfen et al., 1990). Activation of G_αs/olf_‐coupled D_1_ receptors stimulates the cAMP/PKA pathway, which in turn regulates a number of downstream effectors, including GluA1 at a PKA‐catalyzed phosphorylation site, that is, S845 (Chao et al., 2002; Mao et al., 2013; Price et al., 1999; Snyder et al., 2000; Swayze et al., 2004; Xue et al., 2014, 2017). In contrast, blockade of D_1_ receptors resulted in a decrease in constitutive S845 phosphorylation in the striatum (Xue et al., 2017; this study). In addition to D_1_ receptors, adenosine A_1_ receptors are notably expressed in striatonigral neurons (Fuxe et al., 2007, 2010). Since A_1_ receptors are coupled to G_αi/o_ proteins, activation of these receptors inhibits the cAMP/PKA pathway, whereas blockade of them leads to an opposite change in the cAMP/PKA pathway. As a downstream target of PKA, S845 phosphorylation is likely regulated by the A_1_ tone. This study provides evidence in favor of this notion. We found that blocking A_1_ receptors with DPCPX upregulated striatal S845 phosphorylation. This seems to support that there exists a tonic inhibitory tone from A_1_ receptors, which inhibits basal S845 phosphorylation in striatal neurons. Moreover, given the colocalization of A_1_ and D_1_ receptors in striatonigral neurons, an A_1_‐D_1_ interaction model has been suggested. That is, two receptors form a balance to modulate the PKA signaling, thereby controlling the outflow of the D_1_/A_1_‐mediated direct pathway in the basal ganglia (Ferre et al., 1994; Fuxe et al., 2007, 2010). Indeed, we found that blocking D_1_ receptors abolished the A_1_ antagonist‐induced S845 phosphorylation, indicating that the D_1_/A_1_ balance controls S845 phosphorylation. Others found that the A_1_ agonist induced a late decrease in phosphorylation of dopamine‐ and cAMP‐regulated phosphoprotein of M(r) 32 kDa at T34 via postsynaptic A_1_ receptors in striatonigral neurons (Yabuuchi et al., 2006). DPCPX and the adenosine receptor antagonist caffeine elevated expression of c‐fos and other immediate early genes and SCH23390 abolished the effect of caffeine in striatonigral neurons (Dassesse, Vanderwinden, Goldberg, Vanderhaeghen, & Schiffmann, 1999). Future studies will elucidate whether the D_1_/A_1_‐regulated S845 phosphorylation occurs specifically in the phenotype of striatonigral output neurons. In addition to the postsynaptic D_1_/A_1_ interaction, blocking presynaptic A_1_ receptors by DPCPX may facilitate local dopamine release (Borycz et al., 2007; Wood, Kim, Boyar, & Hutchison, 1989). The released dopamine may stimulate D_1_ receptors in striatonigral neurons to upregulate GluA1 S845 phosphorylation in these neurons.

A_1_ receptors are expressed in the mPFC with a high level in pyramidal spiny output neurons (Rivkees, Price, & Zhou, 1995). Ultrastructural analysis reveals the presence of A_1_ receptors in both the presynaptic terminals and the postsynaptic structures (Ochiishi, Chen, et al., 1999). In the present study, we found that blocking A_1_ receptors with DPCPX elevated GluA1 S845 phosphorylation in the mPFC. Thus, A_1_ receptors in mPFC neurons seem to maintain an inhibitory tone on GluA1 S845 phosphorylation. Additionally, the dopamine D_1_ receptor is one of principal dopamine receptor subtypes expressed in pyramidal neurons throughout mPFC layers (de Almeida, Palacios, & Mengod, 2008; Dembrow & Johnston, 2014). Thus, A_1_ and D_1_ receptors in mPFC neurons like in striatal neurons may converge on the cAMP/PKA pathway to control activity of PKA substrates such as GluA1. Indeed, D_1_ receptor activity is required for the A_1_ antagonist‐induced S845 phosphorylation since the D_1_ antagonist SCH23390 abolished the response of S845 phosphorylation to DPCPX in the mPFC.

The hippocampus is among brain regions expressing a high level of A_1_ receptors. In this structure, strong expression of A_1_ receptors was present on cell bodies and dendrites of pyramidal and granule neurons and mossy fibers, but not on glial cells (Ochiishi, Chen, et al., 1999; Ochiishi, Saitoh, et al., 1999; Rivkees et al., 1995). At the subsynaptic level, A_1_ receptors are distributed pre‐ and postsynaptically (Ochiishi, Chen, et al., 1999; Rebola, Pinheiro, Oliveira, Malva, & Cunha, 2003), indicating the importance of A_1_ receptors in modulating both presynaptic neurotransmitter release and postsynaptic physiology. To determine the role of A_1_ receptors in regulating GluA1 phosphorylation in hippocampal neurons, we found that the A_1_ antagonist upregulated S845 phosphorylation in the adult rat hippocampus in vivo. Early studies found that A_1_ receptors physically interacted with GluA1 subunits in hippocampal neurons (Chen et al., 2014). In rat hippocampal slices, prolonged A_1_ receptor activation with CPA reduced GluA1 phosphorylation at S845 and S831 and induced GluA1 endocytosis and synaptic depression (Chen et al., 2014; Stockwell et al., 2016). These data suggest the hippocampus as another brain region where a significant inhibitory tone of A_1_ receptors on S845 phosphorylation exists. Moreover, dopamine D_1_ receptors are critical for the A_1_‐mediated inhibition of S845 phosphorylation in this area. Pretreatment with the D_1_ antagonist blocked the A_1_ antagonist‐induced S845 phosphorylation in the hippocampus.

Changes in S845 phosphorylation are thought to have a significant impact on the subcellular expression pattern and function of GluA1‐containing AMPA receptors. Previous studies found that S845 phosphorylation enhanced the surface trafficking of GluA1 (Man, Sekine‐Aizawa, & Huganir, 2007; Oh, Derkach, Guire, & Soderling, 2006) and AMPA channel peak current (Roche et al., 1996). Since the AMPA receptor‐mediated glutamatergic transmission plays a pivotal role in the regulation of neuronal and synaptic activities in all the forebrain regions surveyed (striatum, mPFC, and hippocampus), the A_1_ receptor‐regulated S845 phosphorylation is believed to be a biochemical step critical for carrying out normal functions of these brain structures, a topic to be investigated in‐depth in the future.

Our studies on AMPA receptor phosphorylation were performed in male rats. While sex differences in the A_1_‐regulated AMPA receptor phosphorylation have not been reported in striatal neurons to our knowledge, it is important to assess the response of AMPA receptors to adenosine receptor agents in female animals in future studies.

## CONFLICT OF INTEREST

The authors declare that they have no conflict of interest.

## Data Availability

The data that support the findings of this study are available from the corresponding author upon reasonable request.
